# Skin Lesion Synthesis and Classification Using an Improved DCGAN Classifier

**DOI:** 10.3390/diagnostics13162635

**Published:** 2023-08-09

**Authors:** Kavita Behara, Ernest Bhero, John Terhile Agee

**Affiliations:** 1 Department of Electrical Engineering, Mangosuthu University of Technology, Durban 4031, South Africa; 2 Discipline of Electrical, Electronic and Computer Engineering, University of KwaZulu-Natal, Durban 4041, South Africa; bhero@ukzn.ac.za (E.B.); ageej@ukzn.ac.za (J.T.A.)

**Keywords:** convolutional neural network, deep learning, skin cancer, generative adversarial network

## Abstract

The prognosis for patients with skin cancer improves with regular screening and checkups. Unfortunately, many people with skin cancer do not receive a diagnosis until the disease has advanced beyond the point of effective therapy. Early detection is critical, and automated diagnostic technologies like dermoscopy, an imaging device that detects skin lesions early in the disease, are a driving factor. The lack of annotated data and class-imbalance datasets makes using automated diagnostic methods challenging for skin lesion classification. In recent years, deep learning models have performed well in medical diagnosis. Unfortunately, such models require a substantial amount of annotated data for training. Applying a data augmentation method based on generative adversarial networks (GANs) to classify skin lesions is a plausible solution by generating synthetic images to address the problem. This article proposes a skin lesion synthesis and classification model based on an Improved Deep Convolutional Generative Adversarial Network (DCGAN). The proposed system generates realistic images using several convolutional neural networks, making training easier. Scaling, normalization, sharpening, color transformation, and median filters enhance image details during training. The proposed model uses generator and discriminator networks, global average pooling with 2 × 2 fractional-stride, backpropagation with a constant learning rate of 0.01 instead of 0.0002, and the most effective hyperparameters for optimization to efficiently generate high-quality synthetic skin lesion images. As for the classification, the final layer of the Discriminator is labeled as a classifier for predicting the target class. This study deals with a binary classification predicting two classes—benign and malignant—in the ISIC2017 dataset: accuracy, recall, precision, and F1-score model classification performance. BAS measures classifier accuracy on imbalanced datasets. The DCGAN Classifier model demonstrated superior performance with a notable accuracy of 99.38% and 99% for recall, precision, F1 score, and BAS, outperforming the state-of-the-art deep learning models. These results show that the DCGAN Classifier can generate high-quality skin lesion images and accurately classify them, making it a promising tool for deep learning-based medical image analysis.

## 1. Introduction

According to research on human anatomy by medical professionals, the skin is the largest and heaviest single biological tissue in the human body, covering roughly 20 square feet in average surface area and 6 pounds in typical weight [1,2]. The skin is the body’s first line of defense, yet it is not entirely impenetrable. As a result, the skin is continually susceptible to diverse environmental and genetic factors. Therefore, skin conditions affect everyone, regardless of age, skin tone, way of life, or socioeconomic status. In a recent study, the Skin Cancer Index 2018 [3] showed that geographic and geopolitical factors make some places more likely than others to have skin cancer, which can occasionally be fatal. Computer-aided systems that automate the diagnosis of skin problems are the focus of research [4,5,6,7,8]. Image acquisition, preprocessing, segmentation, feature extraction, and classification methods have been developed. Non-standard cameras, cell phones, and digital cameras can produce medical images [9,10].

Skin cancer is one of the most lethal forms of cancer. Mutations in the skin’s genome, created by damaged or uncontrolled growth of cells in the skin, cause skin cancer. Skin cancer tends to progressively metastasize to other anatomical sites, rendering it more amenable to treatment during its early stages [11]. Consequently, early detection is paramount in optimizing the chances of effective treatment and a higher chance of survival. The growing incidence of skin cancer cases, elevated death rate, and costly medical interventions necessitate early detection of its symptoms [12]. Given the significance of these concerns, researchers have developed several automated diagnostic systems for early skin cancer diagnosis. Lesion criteria, including but not limited to symmetry, color, size, and form, are considered in identifying skin cancer and the differentiation between benign skin cancer and melanoma [13]. However, automatically classifying skin cancer has significant challenges due to a scarcity of diverse, annotated, and high-quality training data for skin disease images. Ensuring the model’s ability to adapt across different domains and maintain robustness is also a vital challenge. Collecting and annotating a large dataset of skin cancer images is often time-consuming and costly, and it may raise privacy concerns. Synthetic data generation techniques can address this issue by creating realistic synthetic images that can be combined with real data to enhance the size and diversity of the training dataset. This process helps train more robust and generalizable models, which are better equipped to handle variations in skin types, lesions, and imaging conditions, ultimately leading to more accurate and reliable skin cancer detection systems [14].

The adoption of artificial intelligence (AI) in medicine is increasing and has the potential to revolutionize clinical treatment and dermatology processes. However, to ensure the final output’s fairness, dependability, and safety, specific criteria-setting development and performance evaluation requirements must be met when developing image-based algorithms for dermatology applications [15,16]. In health informatics, ISO 12052:2017 addresses the transfer of digital images and data of their creation and management between medical imaging equipment and systems handling the management and transmission of that data [17].

Diagnostic imaging, such as CT scans, MRIs, dermoscopy, radiography, and ultrasound, is often used in medical diagnosis. Medical images enable physicians to diagnose patients more effectively, accurately, and consistently by exhibiting the patient’s internal anatomy and helping identify potential problems. Dermoscopy is most commonly used to identify skin lesions. Dermoscopic images benefit from bright lighting and a low noise level [18]. Therefore, applying deep learning techniques can speed up the development of image-assisted medical diagnostics [19,20]. Deep learning has facilitated the resolution of complicated learning problems previously intractable to rule-based methods [21]. Deep learning-based algorithms have come close to matching human performance on various challenging computer vision and image classification tasks. In [22], it was demonstrated that deep learning systems matched the results of skin cancer classification performed by 21 certified dermatologists.

Several computerized systems using deep learning for diagnosing skin cancer have been proposed [21,22]. A robust and reliable collection of dermoscopic images is necessary to assess their diagnostic performance and validate predicted results. Numerous datasets on skin cancer have lacked size and diversity. Training models for skin lesion classification are hindered by the lack of diverse data and the small size of the datasets. Automated skin cancer diagnosis has predominantly focused on diagnosing melanocytic lesions, resulting in limited diagnoses in available datasets. Consequently, a reliable, standardized dataset of dermoscopic images is crucial.

The International Skin Imaging Collaboration (ISIC) is a university-industry partnership that promotes using digital imaging systems to reduce the number of deaths from melanoma. Teledermatology, clinical decision support, and automated diagnosis use digital skin lesions images to teach specialists and people about melanoma and help diagnose it [23]. ISIC promotes digital skin imaging standards and engages dermatologists and computer scientists to improve diagnosis. Lack of dermatologic imaging standards degrades skin lesion imaging. ISIC proposed digital skin image quality, confidentiality, and interoperability standards (i.e., the capacity to transfer visual data between different clinical and technological systems) [23]. ISIC is creating dermatological and computer science resources, including a vast and expanding open-source public-access skin picture database (Goals of the Archive). This repository provides images for research, study, and testing of diagnostic AI systems. ISIC engages stakeholders through meetings, papers, conferences, and AI Grand Challenges [23]. Classification and segmentation are the most common uses for ISIC datasets. To train their algorithms, researchers focus primarily on binary classification problems. Researchers began experimenting with multiclass classification after the release of ISIC 2018 and ISIC 2019, with the ISIC 2020 dataset as the primary resource. However, melanoma detection was the main goal of the ISIC 2020 challenge. As a result, more research on binary classification can be done. ISIC did not extend this challenge category beyond 2019, suggesting that segmentation tasks are less popular than lesion diagnosis. Segmentation masks were only available for a small subset of datasets (ISIC 2016–2018) compared to most classification tasks. Color constancy research and generative adversarial network-based data enhancement are further applications [24] that use ISIC datasets. Variations in the metrics for image acquisition at the whole-body, regional, close-up, and dermoscopic levels can affect the quality and validity of skin images. Universal imaging standards in dermatology require the establishment of consensus norms. Clinical practice information exchange, documentation in electronic health records, harmonization of clinical trials, database building, and clinical decision assistance all benefit from standard procedures like Delphi for image standardization [25].

Applying deep learning to medical image-assisted diagnosis still presents numerous challenges. A common issue in healthcare applications is a scarcity of adequately diverse, large training datasets. These datasets typically exhibit severe class imbalance [26], which can lead to biased models [27]. A class imbalance occurs when there are disproportionately many samples of one pathology compared to another. As such, networks frequently overfit [28] and fail to generalize to new models [29]. Although more data is available online, most of it is still unlabeled. Annotating medical data is time-consuming and expensive as it requires specialized diagnostic tools and expert clinicians, which is why it is one of the critical challenges to generating models suitable for clinical application.

These substantial restrictions result in two major issues: (1) restricted generalization, biased datasets, overfitting, and data convergence; and (2) precise classification. To address the first problem, the authors proposed an improved Deep Convolution Generative Adversarial Networks (DCGANs) classifier approach to generate convincing synthetic data for training skin lesion classification models with improved generalization and to prevent the model from overfitting [30]. Skin lesions are treated using effective image filtering and enhancement methods to improve the model’s ability to find and extract features during training. As a solution to the problem of scarce medical datasets, basic GANs and networks based on GANs that generate synthetic medical images have gained significant traction in recent years. GANs [31] attempt to mimic the distribution of actual images by making the synthetic samples look precisely like real images, thereby increasing the Classifier’s capacity to distinguish between the skin types. Generator-Discriminator networks are used to generate synthetic skin lesions using dermoscopic features. Synthetic images of skin lesions using multiple modalities could solve the problem and give deep learning models more and better data. Several medical algorithms [32] rely heavily on these features, which are local patterns in the lesion. To address the second issue, we used a stack of convolution layers in the discriminator network, which acts as a classifier for class prediction. Our proposed framework demonstrates that DCGAN augmentation improves performance, which is critical in high-stakes clinical decision-making, although it requires an additional trained network compared to typical augmentation procedures. This study investigated the prospect of improving Deep Neural Network diagnostic performance by incorporating DCGAN data into training data.

The main contribution to this study is as follows:

1. Construct and train an improved DCGAN classifier using customized synthetic augmentation techniques and fine-tune the parameters for skin lesion classification that can accurately diagnose skin lesions;
2. Investigate whether the synthetic images generated by a multi-layered convolutional generative network accurately reflect the distribution of the original image dataset. In contrast, a discriminator perceptron, which is also multi-layered, tries to distinguish between false and real image samples;
3. Evaluate the performance of the improved DCGAN Classifier compared with existing state-of-the-art classifiers for skin lesion classification.

The rest of the work is organized as follows: Section 2 discusses the related topics; Section 3 provides the methods used for the model; the results and discussion of the performed experiments are described and discussed in Section 4; and finally, the conclusion is presented in Section 5.

## 2. Related Work

Variations in skin tone, illness location, and picture capture devices complicate the development of automated skin disease detection systems. Furthermore, skin issues have distinctive characteristics, making classification difficult. However, CNN has achieved remarkable success in the medical industry as well as in other fields, providing optimism for the future of automated medical system development [33,34].

The generative adversarial network (GAN) is another technique that has researchers intrigued because of its ability to represent complex real-world visual input. It can also normalize unbalanced data sets [35]. However, few applications have used GAN beyond simple binary classification and data augmentation [36].

Researchers commonly use data augmentation strategies to boost the models’ robustness and generalizability [33,34,35,36]. Standard augmentation techniques for enhancing image data include resizing, rotating, flipping, and shifting the original image. These traditional data augmentation techniques have become commonplace in computer vision network training [37,38]. Unfortunately, these techniques are limited in their ability to yield important new insights from relatively few adjustments to the source data.

Data synthesis, a more advanced data augmentation method [39], shows promise and is the subject of much interest. Among the many approaches for creating synthetic data, variational autoencoders (VAEs) [40] and generative adversarial networks (GANs) [41] stand out as the most common. The latent representation of VAEs is highly structured and continuous, making them simple to train. Despite their challenging and unreliable training procedures, GANs can produce high-quality, realistic images. Despite the success of GANs in medical imaging [42,43], there has been little systematic investigation into the synthesis of images of skin lesions and class imbalance [44]. By investigating the issue of class imbalance across various data regimens and contrasting the effectiveness of conventional and GAN data augmentation methods, we seek to close this gap.

Academics have developed several variants of GANs to enhance their capacity to generate images. The authors of [45,46] integrated a convolution operation into a Deep Convolutional Generative Adversarial Network (DCGAN) to improve GAN performance. Moreover, to enhance the diversity of generated images, the noise vector is sampled by DeLiGAN and fed into the Generator as noise [47].

In [48], authors combined meta-learning and CGAN to propose MetaCGAN, a new variation of GAN. MetaCGAN can transfer its knowledge from large dataset training to a new problem with a small dataset. In recent years, some researchers have created high-resolution images using convolutional and recurrent neural networks (CNNs and RNNs). However, the algorithms produce images one pixel at a time rather than in their entirety [49,50]. In [51], categorical generative adversarial networks using catGAN [52] and Wasserstein distance [53] are presented. CatWGAN can identify input data and generate 64 × 64-pixel images. Using a dataset of 140 labeled images from the ISIC 2016 Challenge, the proposed method outperformed the denoising autoencoder and the simple hand-crafted features with an average precision of 0.424 [54]. Using the Pseudo Cycle consistent module and the domain control module, a variant of Cycle GAN was introduced in [54] to generate CT images. The domain control module supplies supplementary domain information, while the Pseudo Cycle consistent module ensures that all created images look identical. Baur et al. [55] proposed deeply discriminated GAN to improve image resolution (DDGAN). The authors created realistic 256 × 256 skin lesion images and compared DCGAN, LAPGAN, and DDGAN [56,57], showing that both can learn dataset distributions and synthesize realistic samples.

The authors [58] also synthesized realistic, high-resolution skin lesion images. Progressive growing of GANs (PGGAN) [59] helped them synthesize images from noise at 1024 × 1024 pixels. The Visual Turing Test and Sliced Wasserstein Distance were used to evaluate DCGAN [60], LAPGAN [60], and PGGAN images. The Visual Turing Test reveals that even trained dermatologists have problems recognizing fake images from real ones. Jiang et al. [61] proposed using a Fused Attentive GAN (FA-GAN) to create and reconstruct high-resolution MR images [61]. Built modules for local and global feature extraction to extract valuable traits. FA-GAN used PSNR and SSIM performance metrics to train the network on 40 sets (256 slices) of 3D MR images. The authors used pix2pixHD GAN for image generation in [62,63] and generated images using semantic and instance mappings. The pix2pixHD GAN is an innovative approach to using meaningful skin lesion knowledge to synthesize high-quality images and improve skin lesion classification performance; however, it does require annotated data to create images. The authors exploited GAN-synthesized breast ultrasound images for breast lesion categorization and augmentation [64].

The study in [65] presented Cascade Ensemble Super-Resolution CESR-GAN to rebuild high-resolution skin lesion images from low-resolution photos. They created an image-based loss function [65]. In [66], the author enhanced the image data of skin lesions using GANs. The GAN discriminator was the final Classifier trained to recognize seven categories of skin lesions from the ISIC 2018 challenge dataset [67,68]. Transfer learning is used to improve the DenseNet [69] and ResNet [70] architectures’ classification performance compared to the GAN-based data augmentation model. The suggested method raised the score for balancing correctness.

One of the most challenging and well-liked research areas is the simulation of medical imaging in many medical specialties. Researchers use intense architectures to enhance their findings. GANs are frequently employed in a variety of medical imaging applications. GAN input noise management in medical imaging still has room for innovation. Medical image synthesis, segmentation, detection, classification, and reconstruction using GANs are becoming increasingly common [71]. However, over the last two years, the image synthesis-based data augmentation technique has been applied for skin lesion imaging. Previous literature searches reveal a few related studies on skin lesion classification. Table 1 summarizes the research needs for determining and analyzing the available literature. Skin lesion image generation requires the further use of specific, more advanced GAN designs that can produce high-resolution representations.

The research gap is identified by analyzing the available literature and is summarized in Table 1. We have investigated DCGAN’s potential on our dataset to contribute to developing a reliable skin cancer detection and classification application.

## 3. Methods

This study proposes a skin lesion DCGAN Classifier model based on a GANs framework that can produce high-quality images of skin lesions. The authors have investigated the potential of DCGAN on the ISIC 2017 dataset to create a reliable tool for detecting and classifying skin cancer.

### 3.1. Skin Cancer Dataset

**Figure 1:**
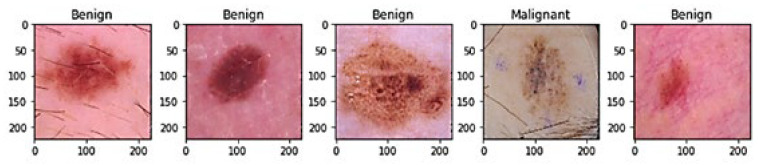
Sample images of benign and malignant.

The first stage in skin lesion classification is acquiring a high-quality dataset to train our proposed model. The ISIC datasets are widely utilized for automated skin lesion analysis due to the need for more high-quality, annotated images of skin lesions. Our DCGAN model was trained using 2000 images from the ISIC 2017 skin cancer dataset, uniformly distributed between benign and malignant cases. When using deep learning to classify skin lesions, gathering an extensive dataset representing a wide range of lesions is essential. Images are typically labeled with the type of skin lesion depicted, providing the algorithm with ground truth. The dataset contains images of varying resolutions. It is a complex task to accurately categorize due to the resolution variability and class imbalance. Figure 1 shows a sample representation of both benign and melanoma skin lesions. During the random selection of test images, the authors tried to maintain a consistent class distribution of the data set and ensure that each class’s percentage of test images was comparable to that of the train data set. For this purpose, the classes in the data were first labeled and annotated as follows:

- Image name: a unique identifier that refers to the filename of the corresponding image;
- Patient ID: a unique identifier assigned to each patient;
- Sex: the gender of the patient or a blank field if unknown;
- Approximate age: the patient’s approximate age at the time the imaging was conducted;
- Anatomical site: the location of the imaged site on the patient’s body;
- Diagnosis: detailed diagnostic information (only included in the training data);
- Benign/malignant indicates whether the imaged lesion is benign or malignant;
- Target: a binarized form of the target variable.

### 3.2. Proposed Framework of the DCGAN-Based Classifier

**Figure 2:**
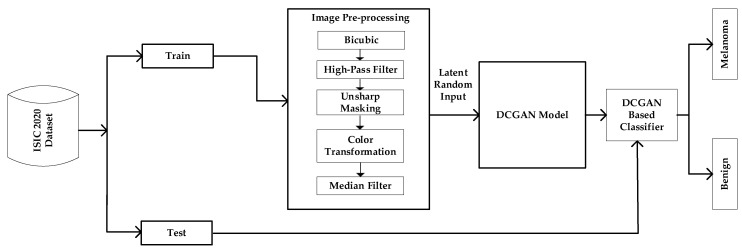
A framework of the proposed GAN-based classifier.

The proposed framework consists of three distinct phases, namely (1) Image Preprocessing, (2) DCGAN Modeling, and (3) Classification, as depicted in Figure 2. Each of these phases is addressed in greater detail in the following subsections. The image Preprocessing methods were performed before the images were fed into the GAN model for classification.

### 3.3. Image Preprocessing Techniques

In image preprocessing, the images are transformed using various preprocessing techniques to reduce noise and other potential artifacts and to put them in a format that can be used for more sophisticated processing. Before being used in image processing, the dataset is divided into train (70%) and test (30%) datasets. The first step in image preprocessing is image scaling, where images are resized to a specified pixel width using the bicubic interpolation method [75,76,77,78,79,80,81]. Each image in the dataset varied in size; therefore, images were scaled to a standard size to improve faster and easier processing. Histogram equalization techniques are used after rescaling the image to improve the image’s intensity values and to modify contrast to increase the brightness of dark images [82,83,84,85,86]. Next, to capture the edges and fine details of the image, the authors used a combination of two image sharpening techniques: Unsharp Masking (USM) [87,88,89] and Gaussian High Pass Filtering (GHPF) [90,91,92], and then color space transformations [90,93,94,95,96,97] were applied to the image. Since color information is essential for skin disease detection systems, we attempted to extract the most relevant color for faster processing. Since the pictures for the model training came from different online dermatology sites and could be different sizes and color spaces, image scaling and color space transformation were used to make all images the same size and color space. All images will be changed to function in RGB space. After analyzing the potential image restoration procedures, the median filter was implemented using Gaussian noise to smooth the images [98,99,100,101,102].

**Figure 3:**
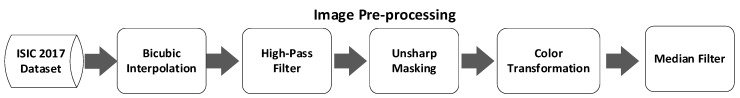
Preprocessing pipeline.

Additionally, it reduces the impact of insignificant factors like fine hairs in pictures of skin lesions. Figure 3 shows the image preprocessing pipeline used in this study. The preprocessed images are fed to the GAN model.

### 3.4. DCGAN Architecture

Deep learning is gaining recognition in image classification because it can extract and choose more features than traditional techniques [102,103]. However, large, labeled datasets are required to maximize deep learning’s potential. Researchers have reported that dermoscopy images are often unlabeled or underlabeled, necessitating expert annotations. GANs are a new and intriguing method that has recently appeared to solve unlabeled datasets [72,73,74]. The ability of GANs to generate precise synthetic data has recently increased their prominence. Also, GANs are seen as a promising approach to the problem of accurately classifying data with low levels of inter-class variation. For learning or sampling complicated probability distributions of image data, GANs are a popular generative model type.

Many researchers have implemented DCGAN in various real-world applications and have produced significantly good results in data synthesis and classification [104]. GANs are prone to instability and might be difficult to train. Finding the ideal balance might be challenging because the training involves a minmax game between the generator and discriminator networks. It is common for GANs to suffer from mode collapse, which occurs when the generator does not accurately reflect the diversity of the target distribution. To address these gaps, the authors in [45,105] proposed Deep Convolutional Generative Adversarial Networks (DCGAN), which have a set of architectural constraints to balance GANs.

DCGAN is an extension of the GAN design that uses deep convolutional neural networks for both the generator and discriminator models, as well as configurations for the models and training that lead to stable training of a generator model. The DCGAN offers the model constraints needed to construct high-quality generator models. This architecture enabled the rapid development of several GAN extensions and applications [74].

The architecture of GANs consists of two multi-layered networks: Generator and Discriminator. Both networks of a GAN are trained simultaneously [72]. The Generator network is tasked with creating synthetic images that closely resemble real images in the training dataset. On the other hand, the Discriminator network seeks to discriminate between real and fake images by evaluating the likelihood that a particular input sample is real or fake [73]. The two networks are trained so that the generator is encouraged to create images that the discriminator finds increasingly difficult to distinguish from real ones. Since GANs may be used to simulate the underlying data distribution, they form the basis of the proposed methodology. As a result, they get better at distinguishing various data sets with minimal alterations. As such, it produces synthetic data for each category to train the discriminator by employing a GAN to anticipate the data distribution. After training, the discriminator can accurately classify brand-new samples in each category, even when they are like examples in other classes [74].

Mathematically, to learn the generator’s distribution $p_{g}$ over the image data represented as *x*, define the input noise vector z with a prior distribution $p_{z}\left(z\right)$, then represent the data mapping to Generator $G\left(z;W_{g}\right)$ as a neural network with parameters $W_{g}$. The discriminator $D\left(x;W_{d}\right)$ outputs a single scalar, where $D\left(x\right)$ is the probability that *x* comes from data rather than $p_{g}$. Discriminator *D* is trained to maximize the likelihood of correctly labeling training examples and *G* samples. At the same time, Generator *G* is trained to mislead the discriminator by attempting to minimize $\log\left(1-D\left(G\left(z\right)\right)\right)$. So, *D* and *G* play the following two-player minimax game with the value function *V* (*G*, *D*) [24,72,73,74]:

(1) $${min}_{G}{max}_{D}V(G,D)=E_{x∼Pdata(x)}[\log(x)]+E_{x∼Pz(z)}[\log(1-D(G(z)))]$$

Theoretically, the discriminator makes a random guess as to whether the inputs are real or fake, and the solution to this minimax game is where $p_{g}=p_{data}$.

**Figure 4:**
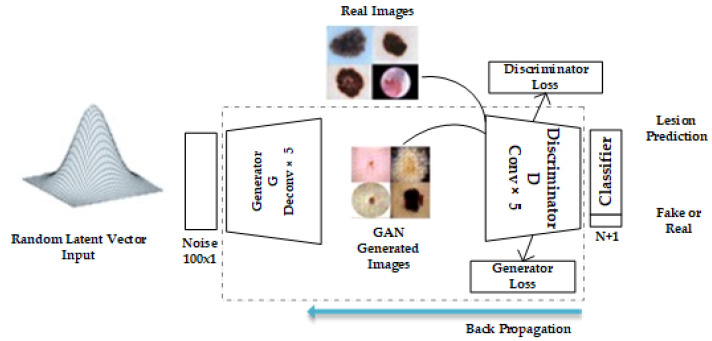
Flow diagram of proposed DCGAN architecture for skin lesion classification.

**Figure 5:**
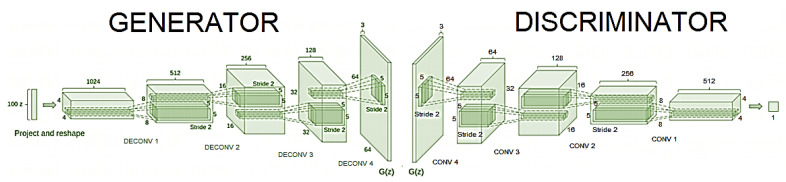
Improved DCGAN framework.

The primary goal of DCGAN is to extend the capabilities of GAN by making use of Convolution Network designs. Radford [45] achieved consistent outcomes by advocating for a few critical architectural limits on DCGAN. The authors of this study employed the basic ideas proposed by Radford [45] to classify skin lesions. Figure 4 and Figure 5 illustrate the flow diagram and improved simulation model used for the DCGAN framework. The following modifications are made to the Generator and Discriminator networks to avoid mode collapse, model instability, and convergence [45]:

Modifications to the Generator:

- The generator uses five deconvolutional layers instead of four;
- Replace deterministic spatial pooling layers such as global average pooling with 2 × 2 fractional-stride convolutions (Generator), which allows the networks to learn by themselves spatial downsampling;
- Eliminate connected hidden layers to avoid model instability and stabilize the convergence speed;
- Update the generator weights using backpropagation and an optimizer SGDM with a constant learning rate of 0.01 instead of 0.0002;
- Batch normalization is used to stabilize the learning of the generator;
- All generating levels use the ReLu activation, except the output layer, which employs the Tanh activation to scale the output between −1 and 1.

Modifications to Discriminator:

- The discriminator uses five convolutional layers to train the networks instead of four;
- Replace deterministic spatial pooling layers such as max pooling with 2 × 2 stride convolutions (the discriminator), allowing the networks to learn spatial upsampling by themselves;
- Eliminate connected hidden layers to avoid model instability and stabilize the convergence speed;
- Update the weights of the discriminator using backpropagation and an optimization step;
- Batch normalization is used to stabilize the learning of the discriminator;
- The LeakyReLU activation function is used for all layers in the discriminator except the output layer to allow gradients to flow backwards through the layer;
- The final layer functions as a classifier and uses the SoftMax activation function for classification.

#### 3.4.1. Model Training and Classification

The authors trained the proposed Improved DCGAN model on the ISIC 2017 dataset to classify the cutaneous skin lesion. Firstly, the dataset is split into training (70%) and testing (30%) datasets. The training dataset is preprocessed and augmented, and then passed into the proposed DCGAN model for training. A preprocessed random latent vector input is fed to the generator to generate synthetic data. The deconvolution neural network of our generator is activated by calling the transposedConv2dLayer function to perform weight multiplication and the Bias function to execute bias addition. In addition to performing weight multiplication and bias addition, the convolution neural network in the discriminator, called the convolution2dLayer function, performs weight multiplication. A transposed convolutional layer is typically used for upsampling or to generate an output feature map with a larger spatial dimension than the input feature map, thereby generating synthetic images. As shown in Figure 5, the authors built the generator to meet DCGAN framework requirements and set OUTPUT_SIZE to 64. The deconvolution step is 2, and each output is four times the input, so each layer’s output size is 4 × 4, 8 × 8, 16 × 16, and 32 × 32, as well as feature maps set to 512, 256, 128, and 64, respectively. The Generator images were rescaled to the range of [−1, 1] for the Tanh activation function. The model was trained with a mini-batch size of 64 using the Stochastic Gradient Descent with Momentum (SGDM) optimizer with a learning rate set to 0.01 and momentum set to 0.5 to stabilize the training. The default values were initialized to zero for all the weights and the normal distribution, with a 0.02% standard deviation.

The discriminator is a feed-forward neural network with four convolutional neural networks. LeakyReLU is set to 0.2. In addition to discriminating between three target classes, the discriminator also functions as a classifier. As a result, the discriminator output layer contains *N* + 1 (=3) units, where n is the number of target classes. The final layer, *D*, is labeled for predicting the probability of an image being real or fake. The remaining *N* units in three layers are trained using a standard cross-entropy loss function, which is represented as [105]:

(2) $$L_{supervised}=-\sum p(D(x)\log(d(D(x))))$$

whereas *D*(*x*) is the correct class label corresponding to the input *x* and $p\left(D\left(x\right)\right)$ is the probability of the predicted class by discriminator *D*. Adding an extra label for the fake class allows us to train the discriminator network across two loss functions simultaneously: one for recognizing fake and genuine images and the other for classification inside actual images. The discriminator network backpropagates discriminator loss to update its weights.

The training step involves changing the network’s weights based on the training data while minimizing the loss function, which measures the difference between the anticipated and real data. During the discriminator training phase, the generator is kept constant. Also, the discriminator is kept constant during the Generator training phase. As discriminator training seeks to determine how to differentiate between real and fake data, it also learns how to identify the generator’s defects. Regarding discriminators, it is assumed that the real input needs to be close to 1 and that the result of the generators will be 0. For the generator network, the discriminator should predict 1 for each image it makes. During training, the validation data assesses the network’s performance and prevents overfitting. Probability distributions are what GANs attempt to reproduce. Therefore, we employed loss functions that account for the discrepancy between GAN’s output and real distribution. The total loss for the discriminator is the sum of the losses for real and fake images [104]:

(3) D_loss=D_real_loss+D_fake_loss

*G_loss* is the cross-entropy resulting from the difference between the generator’s generated data and the discriminator’s input data. In our study, the discriminator also serves as a classifier to distinguish between benign and malignant cancers. The mathematical description of a classifier with a training algorithm is shown below (Algorithm 1):
**Algorithm 1** Training the Classification Model Based on DCGAN-Based ClassifierInput: 1: Load the dataset ISIC2017_Training_Data, *S*17; 2. Split the dataset: Training 70% and Testing 30% 3: Preprocessing of *S*17: *interp2()*, *histeq()*, *imsharpen()*, *imfilter()*, *rgb2lab()*, *gaussian_median_filter()* 4: Initialize the networks: Generator *G(latent_noise)*, Discriminator *D* (); 5. Create optimizers to update the weights using backpropagation *sgdmupdate () and learning rate()*; 6. Train the networks *G* with noise and *D* with real and G-generated images for a number of epochs.    $min_{G}max_{D}V\left(G,D\right)=E_{x\sim Pdata\left(x\right)}\left[\log\left(x\right)\right]+E_{x\sim Pz\left(z\right)}\left[\log\left(1-D\left(G\left(z\right)\right)\right)\right]$ 7. ReLu and Tanh activation function for *G*; Leaky ReLu and SoftMax for *D*; 8. Calculate the loss function and repeat 5–7.          D_loss=D_real_loss+D_fake_loss 9. *D* acts as Classifier *N* + 1 output Output: 1: *N* + 1 Output 2: Confusion Matrix of Classification; Plots of AUC_ROC. 3: return Accuracy, Recall, Precision, Specificity and F1_Score.

## 4. Experimental Results and Discussion

In this section, experimental tests were carried out, including quantitative evaluation of GANs and classifier evaluation, to assess the performance of the proposed skin lesion DCGANs in image synthesis and their application to image classification. The studies used procedural preprocessing processes such as image scaling using the bicubic interpolation approach, normalization, which normalizes image pixel values to the range [0, 1], Sharpening techniques, color transformation, and median filters. The study is performed on an Intel Core i7 processor running at 4 GHz with 12 GB of GPU RAM from an NVIDIA K80 GPU with 4.1 TFLOPS of performance.

### 4.1. Performance Metrics

The authors employed qualitative and quantitative evaluation measures to assess the effectiveness of the DCGAN model. In the qualitative approach, the authors focused on the quality of the image, and in the quantitative overfitting approach, diverse images and mode-dropping problems were evaluated. In this study, evaluation is done in two phases: (1) the Image Preprocessing phase and (2) the Classification Phase. In the Image Preprocessing phase, Mean Square Error (MSE) [105], Peak Signal-to-Noise Ratio (PSNR) [105] and Structural Similarity Index (SSIM) [106] were used. To assess the classification performance of the model, Balanced Accuracy Score (BAS), accuracy, recall, precision, specificity, and F1-Score [74,104,105,106,107] were used as evaluation metrics, which are developed from True Positive (TP), True Negative (TN), False Positive (FP), and False Negative (FN) predictions. The confusion matrix and AUC-ROC plot are used for the graphical presentation of the accuracy of the proposed improved model [108].

In a binary classification problem, there are only two outcomes that can be either positive or negative. The key metrics in medical diagnosis are recall and specificity, which are used to compute BAS. Specificity refers to the instances of positives, whereas recall refers to the instances of negatives. The Balanced Accuracy Score (BAS) is a metric used to assess a classifier’s performance on imbalanced datasets. It is determined by averaging the Classifier’s sensitivity and specificity. The ROC curve illustrates the compromise between sensitivity and specificity (false positive and false negative rates) as a function of the threshold value. The proportion of real negatives incorrectly identified as positives is known as the false positive rate. A contrast to this is the actual positive rate, which measures how successfully positive samples are recognized. The ROC figure compares different thresholds in terms of the true positive rate (*y*-axis) and the false positive rate (*x*-axis). The curve of a random classifier, depicted as a diagonal from left to right, has 50% sensitivity and 50% specificity. A perfect classifier, however, would have a ROC curve that intersects the upper left corner of the plot with a sensitivity and specificity of 100%. The area under the curve (AUC) measures the Classifier’s overall efficacy. An AUC of 1 indicates an ideal classifier, while an AUC of 0.5 indicates an unpredictable classifier. The Classifier’s performance is generally better the closer the AUC is to 1 [104].

### 4.2. Results of Image Preprocessing Techniques

This section presents the experimental results of the image processing phase. MSE, SSIM, and PSNR evaluation methods are used to measure the quality of the image.

#### 4.2.1. Image Scaling

**Figure 6:**
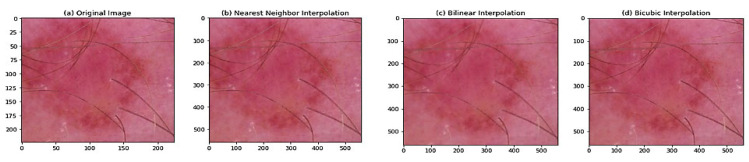
Three methods of interpolation for rescaling the image.

In this study, we have used the bicubic interpolation technique to accurately assess a pixel’s intensity by extrapolating from neighboring pixels’ values. We compared the image produced by the bicubic interpolation with the other image scaling techniques, namely, nearest neighbor and bilinear interpolation, as shown in Figure 6. As bicubic interpolation determines the value of each pixel by taking the weighted average of the 16 most relative neighboring pixels, the output of bicubic algorithms is smoother and more accurate in preserving tiny features from the input image. As shown in Figure 6 and Table 2, bicubic yields significantly sharper images compared to the other two techniques and represents the optimum balance between processing speed and output quality.

#### 4.2.2. Histogram Equalization

**Figure 7:**
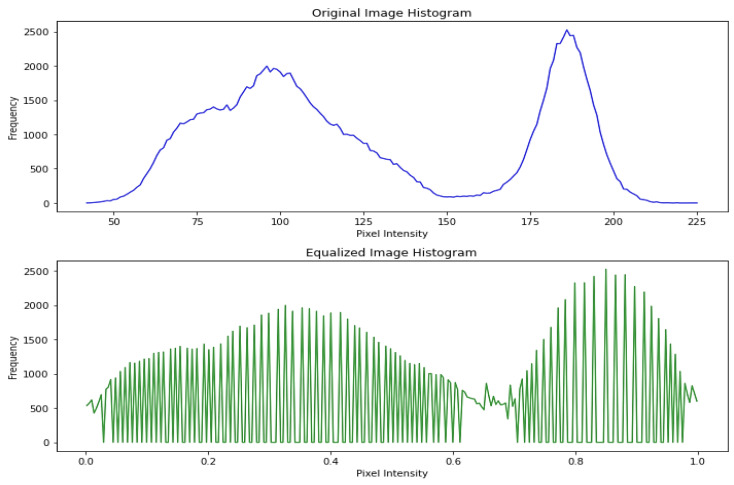
Distribution of pixel intensities.

After bicubic interpolation, Histogram Equalization is performed to increase the contrast of an image. Contrast is achieved by effectively distributing the most commonly occurring intensity values, which stretches the intensity range of the image. This method often increases the overall contrast of the images, which is helpful when close contrast levels indicate meaningful data. It makes it possible for areas of lower local contrast to improve their contrast. Normalization is performed by dividing the histogram by the number of pixels in the analyzed image. To assess the performance of a histogram equalizer, we analyzed histograms before and after equalization to see how the distribution of pixel intensities changed, as shown in Figure 7.

**Figure 8:**
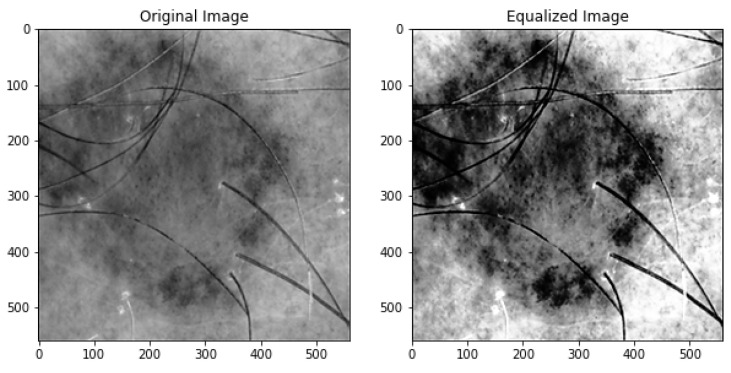
Equalized image histogram.

**Figure 9:**
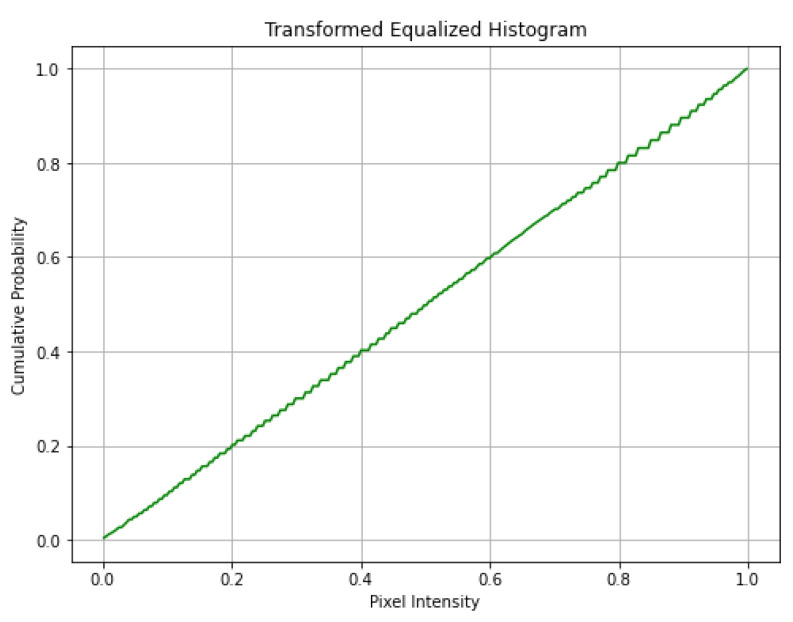
Transformed equalized histogram.

Figure 8 displays the outcome of applying histogram equalization to the low-contrast test image. After adjusting the contrast and brightness, the equalized image’s histogram was computed. Then, the equalized images were normalized by the cumulative sum of the histogram values to generate the cumulative distribution function (CDF). Finally, the equalized image CDF is plotted. The pixel intensity values are on the *x*-axis, while the cumulative probability is on the *y*-axis. Figure 9 demonstrates the distribution of pixel intensities across the image and provides an overview of the transformation caused by histogram equalization.

#### 4.2.3. Unsharp Masking and Gaussian High-Pass Filtering

**Figure 10:**
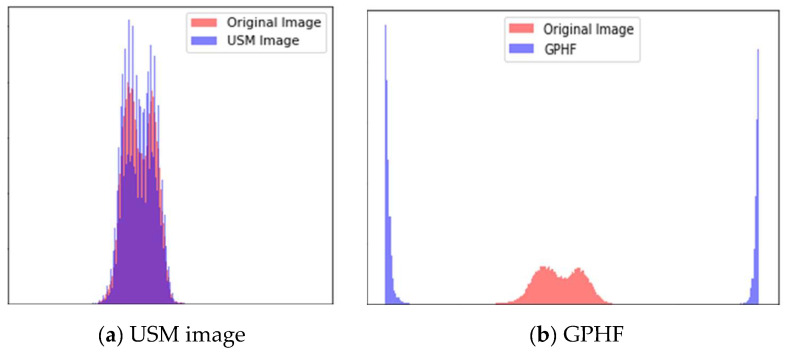
Histogram comparison.

**Figure 11:**
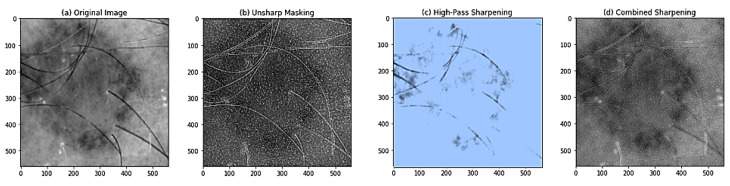
Image comparison between Original, USM, GHPF, and combined sharpening.

We performed the USM and GHPF filtering techniques on the equalized image to sharpen the images and capture the fine details of skin lesions. The authors first used the USM method to enhance the image’s boundaries and fine details, followed by the GHPF method to separate and sharpen the image’s high-frequency values. Figure 10 illustrates the histogram comparison between the original image, the enhanced USM, and the GPHF. The image contrast is adjusted with a histogram equalization method. Then the enhanced images of USM and GHPF are added and multiplied by the weight factor of the image. Figure 11 shows the enhanced, combined, sharpened image. The combined image shows increased visibility of skin lesions and sharpens the image frequency value.

#### 4.2.4. Color Space Transformation

**Figure 12:**
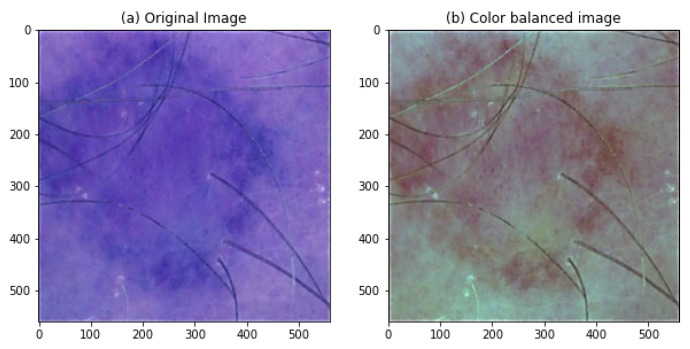
Balanced color space image transformation.

We used the CIELAB transformation to match numerical value changes to perceived color changes. The CIELAB color balance approach preserves color by choosing each channel’s scale factor separately. The first step is to convert each uint8 image to float32 format, map it to LAB color space, split the channels, and calculate the mean and standard deviation along the first axis. The mean and standard deviation determine each color channel’s scale factor. Scaling factors are then added to each color channel along the first axis. After that, reduce the pixel values to make the color channels 0–255. After that, uint8-format the image. Figure 12 shows the color balance transformation, and Table 3 presents the image’s performance after applying the color transformation.

#### 4.2.5. Median Filter

**Figure 13:**
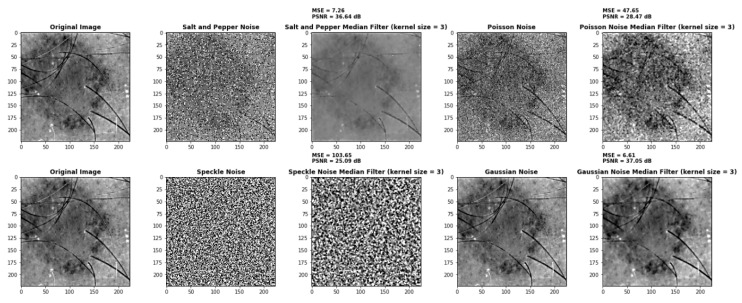
Implementation of median filter on various noises.

In 2D images, the median filter is widely used to eliminate noises such as salt and pepper, speckle, Poisson, and Gaussian noise [96]. Figure 13 shows the implementation of the median filter on these noises. The Mean Square Error (MSE) and Peak Signal Noise Ratio (PSNR) metrics have been used to evaluate these noises. MSE is used to determine whether there is unexpected noise in the image. The value of MSE should generally be low. If so, it suggests that a filter is the best for reducing noise. As illustrated in Figure 13, the MSE values are compared to select the most effective noise-reduction filter. From Table 4, we can conclude that the median filter is best for removing Gaussian noise.

### 4.3. Results of Improved DCGAN-Based Classifier

The ISIC 2017 dataset is used to train the proposed DCGAN-based Classifier. Image preprocessing techniques are applied before DCGAN modeling to ensure the model can learn reliable representations. As discussed in Section 3.4.1, 70% of the original datasets were used for training and 30% for testing each image class. Notably, 30% of the images were taken as unlabeled data. The adversarial network inputs random noise and provides the discriminator’s final prediction on the created images. By altering the noise vector, we may understand how the generator works and which noise vector results in the desired class. However, we have used 100 random latent input noises.

**Figure 14:**
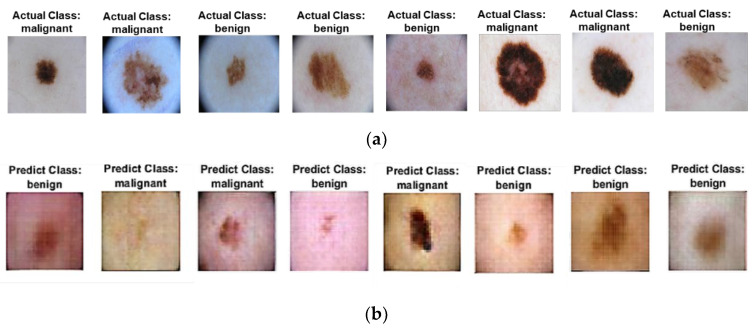
(**a**) Real images from the ISIC 2017 dataset; (**b**) Synthetic images generated by generator.

During the batch normalization, a mini-batch size of 64 was used. All weights were initialized with a normal distribution with a mean of zero and a standard deviation of 0.02. Both the generator and discriminator networks are composed of five layers. The generator comprises four deconvolutional layers with ReLu activations and one deconvolutional layer with Tanh activations at the final layer. The discriminator consists of four convolutional layers with Leaky-ReLu activations and a SoftMax layer in the last layer. During the training process, both actual data and data from the generator network are fed to the discriminator. The generator produces incredibly realistic images after a few epochs of training, which are also used for additional training. To verify the Improved DCGAN model’s effectiveness, we randomly generated 100 images for each of the two skin lesions and compared them to discriminate between real and fake using the BAS evaluation metric; the sample images are shown in Figure 14b. Figure 14a presents the original images from the training data, and Figure 14b shows the random synthetic images generated after training. It is observed that each image produced by DCGAN looks very real and is hard to differentiate from the real images.

**Figure 15:**
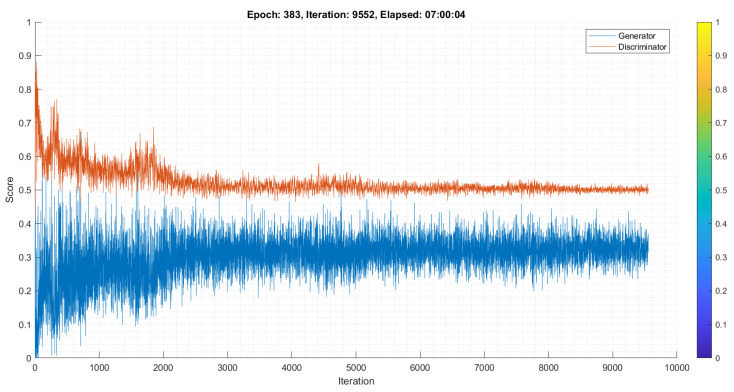
DCGAN generator and discriminator score for each iteration.

Figure 15 shows the DCGAN model’s training process and the score generated by the generator and discriminator after 383 epochs/9552 iterations. The score must be between 0 and 1; however, 0.5 is the best score for each iteration [106]. After each cycle, training losses for generator G and discriminator D are recorded. The generator should get massive random noise early in training to learn how to generate real data. At the same time, the discriminator can distinguish real from fake images; it does not usually collect large samples early on. The generator and discriminator may overcome each other during training. If the discriminator gets too accurate, it will return values close to 0 or 1. If the image generator method is used for producing and classifying skin lesions during training, GAN becomes too accurate; it will constantly harness discriminator mistakes, producing unpleasant effects. In our experiment, the discriminator’s score is close to 0.5, indicating that the generator creates a synthetic image that makes it impossible for the discriminator to differentiate whether it is real or fake.

**Figure 16:**
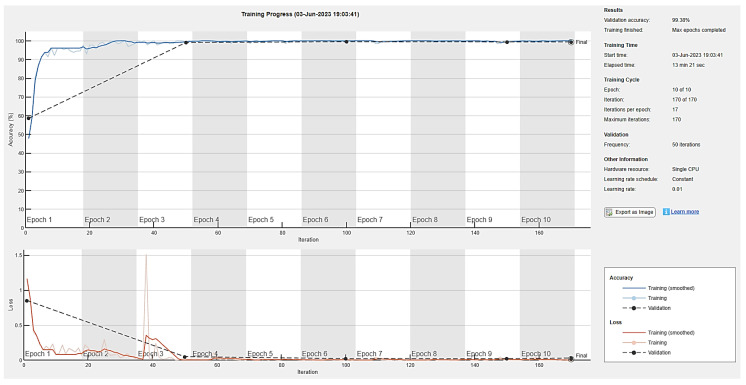
DCGAN training accuracy and loss with a learning rate of 0.01.

**Figure 17:**
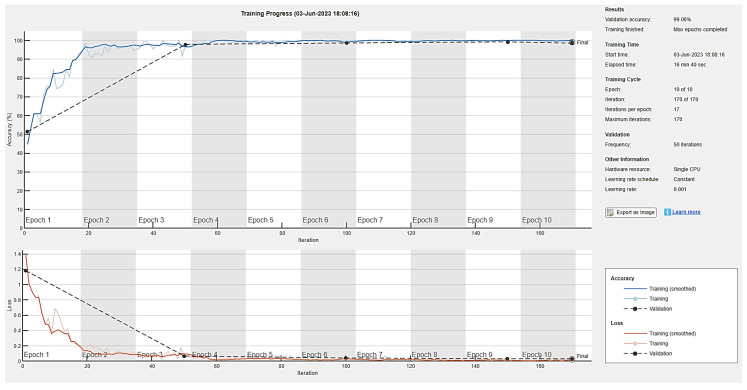
DCGAN training accuracy and loss with a learning rate of 0.001.

**Figure 18:**
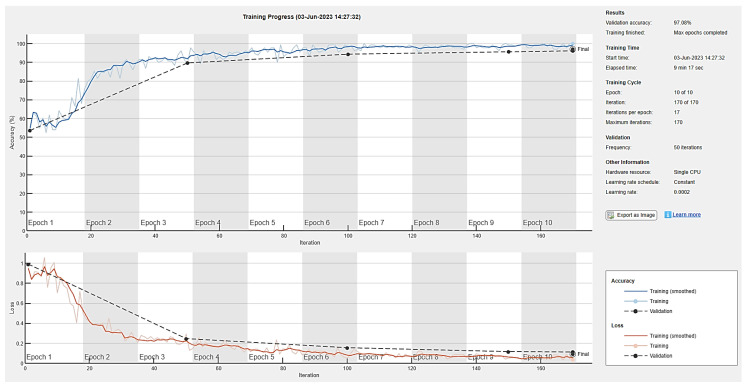
DCGAN training accuracy and loss with a learning rate of 0.0002.

The generator and discriminator networks were trained for ten epochs to examine the validation accuracy of the model with definite learning rates of 0.01, 0.001, and 0.0002. Seventeen iterations per epoch were taken during the training phase, while a validation frequency of 50 iterations was used for testing. Accuracy and loss during training for the generator and discriminator are depicted in Figure 16, Figure 17 and Figure 18 for ten epochs. The loss convergence at the end demonstrates that the GAN model has found its optimal state. Compared to the learning rates of 0.001 and 0.0002, the proposed DCGAN model achieves an optimal accuracy of 99.38% with a learning rate of 0.01, which outperforms state-of-the-art methods of GAN models as suggested in the literature, as depicted in Figure 16, Figure 17 and Figure 18. The images were trained using a single GPU to accelerate the matrix operations, training and parallel computation. Table 5 shows the time elapsed, validation accuracy, and loss of three learning rates computed for ten epochs with a batch size of 64. In modern deep-learning models, batch size is one of the most important hyperparameters to fine-tune the model’s performance. To enable the model to detect the pattern in the data without having to train on a huge dataset, the authors additionally evaluated the model on 128 and 256 batch sizes with a learning rate of 0.01, which gives higher accuracy than 0.001 and 0.0002.

The learning rate is a modifiable hyperparameter employed in training neural networks. Hence, it is imperative to examine the impact of the learning rate on the performance of a model, which is typically assigned a small positive value within the interval of 0.0 to 1.0. The learning rate parameter controls how the model is modified or adapted to the specific problem. A large learning rate allows the model to learn faster but may lead to suboptimal weights and oscillating performance. The model’s loss on the training dataset may fluctuate, and the weights may diverge. On the other hand, a smaller learning rate may help the model reach a more optimal or globally optimal set of weights. Still, it may take significantly longer to train, potentially getting stuck on a suboptimal solution if the learning rate is too small. Therefore, a learning rate of 0.01 typically works for standard multi-layer neural networks and is trained effectively using stochastic gradient descent with momentum (sgdm) optimization algorithms. The momentum enhances the optimization process rate, increasing the probability of identifying the best set of weights within a reduced number of training epochs. The learning rate of 0.01 was chosen because it balanced the need for accuracy and stability. The training results showed that the model could learn a good set of weights with a learning rate of 0.01 and that the model was also stable and did not overfit the training data.

Table 6 demonstrates that the training time for a model is substantially reduced as the batch size increases.

**Figure 19:**
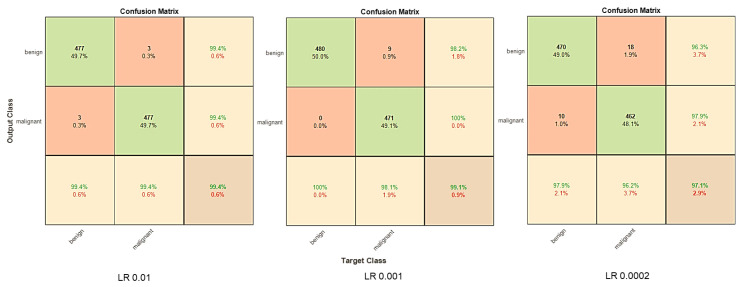
DCGAN confusion matrix.

A confusion plot is a graphical representation of a classification model’s performance. To evaluate model performance, it calculates AUC-ROC, accuracy, precision, recall, and F1 score metrics. As shown in Figure 19, the discriminator successfully diagnoses benign and malignant skin lesions with an extremely high degree of accuracy, as demonstrated in the diagonal elements of the metrics. Figure 19 compares the confusion matrix for three learning rates.

**Figure 20:**
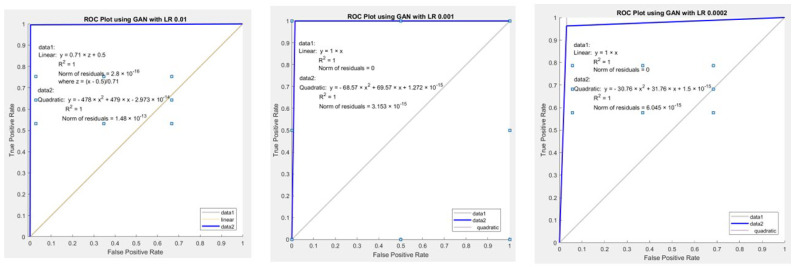
DCGAN AUC-ROC plot.

The ROC curve in Figure 20 summarizes and displays the binary classification results, particularly for the positive class, and has learning rates of 0.01, 0.001, and 0.0002, respectively. We used True Positive (TP) and False Positive (FP) to plot the ROC curve, as mentioned in Section 4.1. TP is on the *y*-axis, and FP is on the *x*-axis in the ROC plot. The plot’s gray line is a random classifier’s ROC curve. The trade-off between sensitivity (TPR) and specificity (1-FPR) is depicted by our ROC curve (Figure 20). Good classifiers perform better if their curves are located nearer the top-left corner. A curve close to the ROC space’s 45-degree diagonal indicates a less accurate test. Despite the ROC curve’s deviation from the diagonal and the little gap between the top left corner and the curve, Figure 20 demonstrates that the Classifier is fairly classified. As shown in Figure 20, data1 is a linear line, and data2 is the ROC curve, a quadratic pattern. R-squared (*R^2^*) for all three learning rates equals 1, indicating that the predicted values are identical to the actual values.

**Figure 21:**
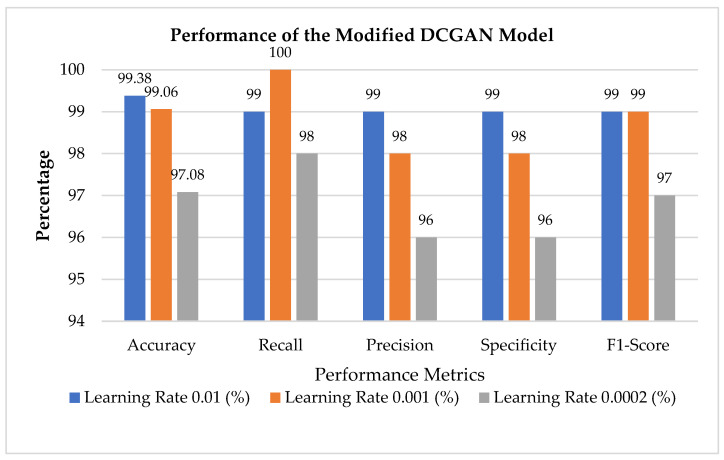
Performance metrics of improved DCGAN.

The performance analysis of the improved DCGAN model utilizing the performance metrics BAS, accuracy, recall, precision, specificity, and F1 Score is shown in Table 6 and Figure 21 for three definite learning rates of 0.01, 0.001, and 0.0002. The model produces good results after 170 epochs, with the test loss reaching a minimal state and the resulting BAS being 99 for learning rates 0.01 and 0.001 and 97 for 0.0002, indicating that the classifier performance is significantly good. The accuracy with a learning rate of 0.01 obtains a greater accuracy of 99.38% compared to a 0.001 model’s accuracy of 98.44% and a 0.0002 model’s accuracy of 96.04%. As a result, the findings indicate that the proposed model could generalize well and operate well when applied to any skin lesions.

### 4.4. Discussion

The results show that the DCGAN method employed in the model’s creation accurately reproduces real skin lesions. A DCGAN-based model allows the creation of more realistic and diverse skin lesions by capturing global structures and detailed textures. Although data augmentation methods like rotation, scaling, and flipping can expand dataset size, the improved strategy goes above and beyond standard methods by synthesizing new lesions. It allows high-quality synthetic samples to be added to missing or unbalanced datasets, improving the model’s ability to generalize to different lesion types. Skin lesions’ enormous diversity and complexity may challenge handmade feature extraction methods such as texture analysis or color-based descriptors, which rely on domain-specific knowledge. The DCGAN Classifier learns and extracts essential features from synthetic skin lesion images automatically, resulting in better generalization, robustness, and accuracy rates, thereby reducing the need for manual feature engineering. The DCGAN-based Classifier can handle differences in lesion appearance, illumination, and image quality due to its improved generalization abilities.

Furthermore, image preprocessing procedures that increased feature extraction and learning were used to improve the accuracy, generalization, and flexibility of the DCGAN-based model. We have used bicubic interpolation, histogram equalization, USM and GHPF, CIELAB color transformation, and a Gaussian noise median filter to extract the feature. In this study, the ISIC 2017 dataset, containing 4000 benign and malignant images, each class containing 2000 images, is used to train and fine-tune the model.

Most effective optimization methods, such as minibatch scholastic, Stochastic Gradient Descent with momentum (sgdm), Adam, Adaboost, etc., are used for fine-tuning hyperparameters [73]. These methods probe the available options to discover the optimal hyperparameter settings for the DCGAN-based classifier. The best-performing hyperparameter combinations were chosen to enhance model performance and generalization. In this study, the authors used a sgdm hyperparameter. To further optimize the model, the learning rate schedule, batch size, activation functions, and number of hidden layers are tuned before the training begins. Hyperparameter optimization, or tuning, in machine learning involves selecting the best hyperparameters for a learning algorithm. Hyperparameters control the learning process. To optimize machine learning, hyperparameters must be tweaked. Hyperparameter optimization identifies a pair of hyperparameters that minimize a loss function on independent data. The traditional methods are grid search, Random search, Gradient-based, Evolutionary, population-based, and early stopping-based [98,100,101,102].

As a result, classification performance is more robust and consistent, even on novel or tough datasets. It can enhance training datasets and allow researchers to analyze sparse or unavailable lesion samples. The DCGAN-based Classifier reliably diagnoses skin lesions, as shown in Table 7 and s 21. It has a high accuracy of 99.38%, 99% for precision, recall, and F1-Score, demonstrating that it can detect and differentiate between different skin lesions. The Classifier is particularly flexible to detect visual contrasts between lesions for precise diagnosis and classification. It solves problems such as class imbalance, annotated data, overfitting, and generalization to new models by capitalizing on DCGANs.

We have compared our proposed model with the existing models in the literature, such as StyleGAN, WGAN, DGAN, SLA-StyleGAN, and DDGAN, in which the GAN classification models were applied to classify the skin lesions as listed in Table 8. These models can also enhance the quality and accuracy of synthetic skin lesions. The improved DCGAN outperforms cutting-edge approaches for synthesizing and categorizing skin lesions. Results show that the proposed improved model is effective at generating accurate predictions based on the test images, and the model attained 99.38% accuracy, which is a significant outcome for skin lesion classification.

Bissoto et al. [29] have achieved a performance of 84.7 using the Pix2Pix GAN model on ISIC 2017. On the other hand, Wu et al. [38] applied GAN with Raman Spectroscopy to augment and generate synthetic images, achieving an accuracy of 92%. Mutepfe et al. [71] have achieved a test accuracy of 93.5% using DCGAN. Qin et al. [72] used StyleGAN and achieved an accuracy of 95.2% and a balanced multiclass accuracy of 83.1%. Khan et al. [73] performed DGAN on unlabeled and labeled datasets, achieving an accuracy of 91.1% and 92.3%, respectively. Therefore, it can be observed that our proposed model achieved a higher accuracy of 99.38% when compared to the models listed in Table 8. These results show that the DCGAN Classifier can generate high-quality skin lesion images and accurately classify them, making it a promising tool for deep learning-based medical image analysis. The model’s accuracy in distinguishing malignant lesions supports early skin cancer detection, enabling timely interventions and improving patient outcomes.

## 5. Conclusions

This study investigated Deep Convolution Generative Adversarial Networks (DCGANs) for their potential application in creating synthetic data for an augmentation technique. This technique has been successfully used to classify images of skin lesions with high accuracy using the ISIC 2017 dataset. Furthermore, the results demonstrate that adding GAN-generated image examples to the training data significantly outperforms conventional approaches to fine-tuning pre-existing deep neural network architectures. Access to unique data generation and augmentation processes like this is helpful when large-scale training datasets are not easily accessible. It enables the addition of high-quality synthetic samples to missing or unbalanced datasets, enhancing the model’s ability to generalize to diverse lesion types. We observed a significant improvement in training after performing image enhancement and preprocessing operations. After fine-tuning the network’s parameters, we obtained an overall test accuracy of 99.38%.

While our model has contributed to improving the performance of skin lesion classification and optimizing diagnostic measures like ROC and other diagnostic test parameters, it is still insufficient to fully validate the software as a clinical diagnostic tool. Although carefully designed to mimic “real conditions”, the computed-generated skin lesions lack complexity and diversity in validating patient data. The proper diagnosis of skin lesions in persons of color is disadvantaged by the absence of dark skin images of pathologic skin lesions in dermatological resources or datasets. Artificial intelligence applications have further harmed people of color because these models are typically trained on images of people with light skin tones. The variation and intricacies present in actual patient samples/images, including varying skin tone backgrounds and subtle differences in malignant and benign skin lesions, cannot be entirely captured by computed-generated images.

This study also had limited capacity to fine-tune its hyperparameters. Conducting all the necessary tests to fine-tune the model took considerable time. The most problematic aspect of this study was the duration of each training session, mainly when training lasted more than 100 epochs. It made it significantly more challenging to optimize the DCGAN on the dataset.

In our future work, we intend to investigate how different lesion-generation approaches can enhance the quality and authenticity of synthetic skin lesions by using larger and more diverse datasets. To improve the precision and dependability of cutaneous lesion classification, we aim to examine how multi-modal fusion with other diagnostic techniques, such as histological information or patient metadata, may be done. Exploring interpretability methods for the DCGAN-based Classifier may provide valuable insights into decision-making and increase reliance on the model’s predictions. Also, Generative model evaluation metrics vary by application. Hence, generative adversarial network evaluation is difficult. Jensen-Shannon divergence, kernel maximum mean discrepancy, and the 1-nearest neighbor classifier can be used to distinguish generated samples from real samples, detect test mode collapsing, and detect dataset-based overfitting by introducing conditional deep convolutional generative adversarial networks. We also aim to investigate parallel processing and distributed training using multiple GPUs to minimize the time required for DCGAN training.

## Figures and Tables

**Table 1:** State-of-the-art methods comparison.

Authors	Techniques	Dataset	Observations	Accuracy (%)
[23]	Pix2Pix GAN	ISIC 2017	The image-to-image translation was done via binary classification using a combination of semantic and instance mappings.	84.7
[38]	GAN with Raman Spectroscopy	Raman Spectroscopy	The authors created a data augmentation module that uses a GAN to generate RS data comparable to the training data classes.	92
[50]	cGAN and WGAN	ISIC 2016	The authors have proposed a categorical generative adversarial network that is both unsupervised and semi-supervised to learn the feature representation of dermoscopy images automatically.	81
[55]	DDGAN	ISIC2017	High-resolution skin lesion synthesis was demonstrated. However, synthetic images were visually low in contrast.	72
[58]	ACGAN, CycleGAN and Path- Rank-Filter	ISIC 2019	Research has proven that random noise and image translation can create high-quality images that look real to the untrained eye. However, these images did not increase the classifier’s performance.	85.6
[63]	DCGAN	ISIC 2016–2021	Conducted a Turing test on the generated images, with 7000 images	58.72
[66]	GAN	ISIC 2018	Created a GAN-based classifier by fine-tuning the existing deep neural architecture.	86.1
[71]	DCGAN	ISIC	The bilateral filter improved training feature recognition and extraction. Fine-tuning the Deep Convolutional Generative Adversarial Network (DCGAN) increased its return. Optimization picked the best network and hyperparameter combinations. Fine-tuning hyperparameter settings takes time and GPU power.	93.5
[72]	styleGAN	ISIC 2018	The generator and discriminator are modified to synthesize high-quality skin lesion images by modifying the generator’s style control and noise input structure. Transfer learning on a pre-trained deep neural network classifies images. Finally, skin lesion style-based GAN synthetic images are added to the training set to improve classifier performance.	95.2
[73]	DGAN	PH2 SD-198 Interactive Atlas of Dermoscopy DermNet	A multiclass technique was utilized to solve the dataset’s class imbalance. Improving the DGAN model’s stability during training has been one of the development’s primary challenges.	91.1
[74]	SLA-StyleGAN	ISIC 2019	The proposed approach outperforms GANs and StyleGANs in key quantitative assessment parameters and quickly produces high-quality skin lesion images. It rebuilds the StyleGAN generator and discriminator structures. Shortcoming Two skin lesions in one photograph might make classification difficult and raise the risk of misdiagnosis.	93.64

**Table 2:** Performance metrics of image interpolation.

	Nearest Neighbor	Bilinear	Bicubic
SSIM	0.88	0.91	0.98
PSNR	31.23	34.62	39.68
MSE	0.0087	0.0089	0.0001

**Table 3:** MSE and PSNR metrics for color space transformation.

	SSIM	PSNR	MSE
CIELAB	0.86	96.92	9.07

**Table 4:** MSE and PSNR metrics for median filter.

	Salt and Pepper Noise	Poisson Noise	Speckle Noise	Gaussian Noise
MSE	7.26	47.65	103.65	6.61
PSNR (dB)	36.64	28.47	25.09	37.05

**Table 5:** Validation accuracy and loss with a batch size of 64.

Learning Rate	Time Elapsed (hh:mm:ss)	Accuracy Minibatch (%)	Validation Accuracy (%)	Mini-Batch Loss	Validation Loss
0.01	00:13:08	100	99.38	0.0007	0.0293
0.001	00:16:27	100	98.44	0.0039	0.0312
0.0002	00:09:17	100	96.04	0.0366	0.1127

**Table 6:** Impact of validation accuracy and loss with different batch sizes.

Batch Size	Time Elapsed (hh:mm:ss)	Accuracy Minibatch (%)	Validation Accuracy (%)	Mini-Batch Loss	Validation Loss
64	00:13:08	100	99.38	0.0007	0.0293
128	00:08:58	100	99.79	0.0040	0.0059
256	00:09:07	100	99.69	0.0003	0.0099

**Table 7:** Performance analysis of improved DCGAN.

Performance Metrics	Learning Rate 0.01 (%)	Learning Rate 0.001 (%)	Learning Rate 0.0002 (%)
BAS	99	99	97
Accuracy	99.38	99.06	97.08
Recall	99	100	98
Precision	99	98	96
Specificity	99	98	96
F1-Score	99	99	97

**Table 8:** Comparison with the existing GAN classification models for skin lesion classification.

Authors	Techniques	Accuracy (%)
[23]	Pix2Pix GAN	84.7
[38]	GAN with Raman Spectroscopy	92
[50]	cGAN and WGAN	81
[55]	DDGAN	72
[58]	ACGAN, CycleGAN and Path- Rank-Filter	85.6
[63]	DCGAN	58.72
[66]	GAN	86.1
[71]	DCGAN	93.5
[72]	styleGAN	95.2
[73]	DGAN	92.3
[74]	SLA- StyleGAN	93.64
Proposed work	DCGAN	99.38

## Data Availability

The experimental datasets used to support this study are publicly available data repositories at https://challenge2020.isic-archive.com, accessed on 20 April 2023.

## References

[1] Hoffman M. (2014). Picture of the Skin: Human Anatomy. https://www.webmd.com/skin-problems-and-treatments/picture-of-the-skin.

[2] Stöppler M.C. (2021). Medical Definition of Skin. https://www.medicinenet.com/skin/definition.htm.

[3] (2018). Skin Cancer-Index 2018. https://derma.plus/en/skin-cancer-index-2018/.

[4] Amarathunga A., Ellawala E.P.W.C., Abeysekara G., Amalraj C.R.J. (2015). Expert system for diagnosis of skin diseases. Int. J. Sci. Technol. Res..

[5] Ambad P.S., Shirsat A.S. (2016). An image analysis system to detect skin diseases. IOSR J. VLSI Signal Process..

[6] ALEnezi N.S.A. (2019). A method of skin disease detection using image processing and machine learning. Procedia Comput. Sci..

[7] Wu H., Yin H., Chen H., Sun M., Liu X., Yu Y., Tang Y., Long H., Zhang B., Zhang J. (2020). A deep learning, image-based approach for automated diagnosis for inflammatory skin diseases. Ann. Transl. Med..

[8] Liu J., Wang W., Chen J., Sun G., Yang A. (2020). Classification and research of skin lesions based on machine learning. Comput. Mater. Contin..

[9] Yan Y., Kawahara J., Hamarneh G. (2019). Melanoma recognition via visual attention. Information Processing in Medical Imaging, Proceedings of the 26th International Conference, IPMI 2019, Hong Kong, China, 2–7 June 2019.

[10] Duan M., Li K., Liao X., Li K. (2018). A parallel multiclassification algorithm for big data using an extreme learning machine. IEEE Trans. Neural Netw. Learn. Syst..

[11] Dildar M., Akram S., Irfan M., Khan H.U., Ramzan M., Mahmood A.R., Alsaiari S.A., Saeed A.H.M., Alraddadi M.O., Mahnashi M.H. (2021). Skin Cancer Detection: A Review Using Deep Learning Techniques. Int. J. Environ. Res. Public Health.

[12] Wu Y., Chen B., Zeng A., Pan D., Wang R., Zhao S. (2022). Skin Cancer Classification with Deep Learning: A Systematic Review. Front. Oncol..

[13] Ali A.R., Li J., Yang G., O’Shea S.J. (2020). A Machine Learning Approach to Automatic Detection of Irregularity in Skin Lesion Border Using Dermoscopic Images. PeerJ Comput. Sci..

[14] Kaur R., GholamHosseini H., Sinha R. (2021). Synthetic Images Generation Using Conditional Generative Adversarial Network for Skin Cancer Classification. Proceedings of the TENCON 2021–2021 IEEE Region 10 Conference (TENCON).

[15] ASRT (2019). The ASRT Practice Standards for Medical Imaging and Radiation Therapy.

[16] (2017). Digital Imaging and Communication in Medicine (DICOM), Including Workflow and Data Management. Health Informatics. https://www.iso.org/obp/ui/#iso:std:iso:12052:ed-2v1:en.

[17] Cassidy B., Kendrick C., Brodzicki A., Jaworek-Korjakowska J., Yap M.H. (2022). Analysis of the ISIC image datasets: Usage, benchmarks, and recommendations. Med. Image Anal..

[18] Sun X., Yang J., Sun M., Wang K. (2016). A benchmark for automatic visual classification of clinical skin disease images. Computer Vision—ECCV 2016.

[19] Srinivasu P.N., SivaSai J.G., Ijaz M.F., Bhoi A.K., Kim W., Kang J.J. (2021). Classification of skin disease using deep learning neural networks with MobileNet V2 and LSTM. Sensors.

[20] Vulli A., Srinivasu P.N., Sashank M.S., Shafi J., Choi J., Ijaz M.F. (2022). Fine-tuned DenseNet-169 for breast cancer metastasis prediction using FastAI and 1-cycle policy. Sensors.

[21] Krizhevsky A., Sutskever I., Hinton G.E. (2017). Imagenet Classification with Deep Convolutional Neural Networks. Commun. ACM.

[22] Esteva A., Kuprel B., Novoa R.A., Ko J., Swetter S.M., Blau H.M., Thrun S. (2017). Dermatologist-level classification of skin cancer with deep neural networks. Nature.

[23] WHO Regional Office for Africa (2013). Handbook for Cancer Research in Africa.

[24] Ibbott G.S., Van Dyk J. (2017). Quality Assurance for Treatment Planning (IEC 62083 and IAEA Report).

[25] Romero-Lopez A., Giro X., Burdick J., Marques O. (2017). Skin lesion classification from dermoscopic images using deep learning techniques. Proceedings of the 2017 13th IASTED International Conference on Biomedical Engineering (BioMed).

[26] Krawczyk B. (2016). Learning from imbalanced data: Open challenges and future directions. Prog. Artif. Intell..

[27] Rice L., Wong E., Kolter J.Z. Overfitting in adversarially robust deep learning. Proceedings of the 37th International Conference on Machine Learning.

[28] Stutz D., Hein M., Schiele B. Disentangling Adversarial Robustness and Generalization. Proceedings of the IEEE/CVF Conference on Computer Vision and Pattern Recognition (CVPR).

[29] Bissoto A., Perez F., Valle E., Avila S. Skin Lesion Synthesis with Generative Adversarial Networks. Proceedings of the International Conference on Medical Image Computing and Computer-Assisted Intervention (MICCAI).

[30] Goodfellow I.J., Mirza M., Xu B., Ozair S., Courville A., Bengio Y. (2014). Generative Adversarial Nets. Adv. Neural Inf. Process. Syst..

[31] Iqbal T., Ali H. (2018). Generative adversarial network for medical images (MI-GAN). J. Med. Syst..

[32] Bi L., Feng D.D., Fulham M., Kim J. (2020). Multi-Label classification of multi-modality skin lesion via hyper-connected convolutional neural network. Pattern Recognit..

[33] Mahbod A., Schaefer G., Wang C., Ecker R., Ellinge I. Skin Lesion Classification Using Hybrid Deep Neural Networks. Proceedings of the ICASSP 2019—2019 IEEE International Conference on Acoustics, Speech, and Signal Processing (ICASSP).

[34] Ayan E., Ünver H.M. Data augmentation importance for classification of skin lesions via deep learning. Proceedings of the 2018 Electric Electronics, Computer Science, Biomedical Engineerings’ Meeting (EBBT).

[35] Zhang J., Xie Y., Wu Q., Xia Y. (2018). Skin Lesion Classification in Dermoscopy Images Using Synergic Deep Learning. Medical Image Computing and Computer Assisted Intervention—MICCAI 2018.

[36] Motamed S., Rogalla P., Khalvati F. (2021). Data augmentation using Generative Adversarial Networks (GANs) for GAN-based detection of Pneumonia and COVID-19 in chest X-ray images. Inform. Med. Unlocked.

[37] Diamant I., Klang E., Amitai M., Goldberger J., Greenspan H. (2018). GAN-based Synthetic Medical Image Augmentation for increased CNN Performance in Liver Lesion Classification. Neurocomputing.

[38] Wu M., Wang S., Pan S., Terentis A.C., Strasswimmer J., Zhu X. (2021). Deep learning data augmentation for Raman spectroscopy cancer tissue classification. Sci. Rep..

[39] Mumuni A., Mumuni F. (2022). Data augmentation: A comprehensive survey of modern approaches. Array.

[40] Razghandi M., Zhou H., Turgut D. Variational Autoencoder Generative Adversarial Network for Synthetic Data Generation in Smart Home. Proceedings of the ICC 2022—IEEE International Conference on Communications.

[41] Yi X., Walia E., Babyn P. (2019). Generative adversarial network in medical imaging: A review. Med. Image Anal..

[42] Kazeminia S., Baur C., Kuijper A., van Ginneken B., Navab N., Albarqouni S., Mukhopadhyay A. (2020). GANs for medical image analysis. Artif. Intell. Med..

[43] Sampath V., Maurtua I., Aguilar Martín J.J., Gutierrez A. (2021). A survey on generative adversarial networks for imbalance problems in computer vision tasks. J. Big Data.

[44] Bissoto A., Avila S. Improving Skin Lesion Analysis with Generative Adversarial Networks. Proceedings of the Anais Estendidos do XXXIII Conference on Graphics, Patterns and Images.

[45] Radford A., Metz L., Chintala S. Unsupervised representation learning with deep convolutional generative adversarial networks. Proceedings of the International Conference on Learning Representations.

[46] Gurumurthy S., Sarvadevabhatla R.K., Babu R.V. DeLiGAN: Generative adversarial networks for diverse and limited data. Proceedings of the IEEE Conference on Computer Vision and Pattern Recognition (CVPR).

[47] Ma Y., Zhong G., Wang Y., Liu W. MetaCGAN: A Novel GAN Model for Generating High Quality and Diversity Images with Few Training Data. Proceedings of the 2020 International Joint Conference on Neural Networks (IJCNN).

[48] Van Den Oord A., Kalchbrenner N., Kavukcuoglu K. Pixel recurrent neural networks. Proceedings of the 33rd International Conference on Machine Learning.

[49] Van Den Oord A., Kalchbrenner N., Vinyals O., Espeholt L., Graves A., Kavukcuoglu K. Conditional image generation with PixelCNN decoders. Proceedings of the 30th International Conference On Neural Information Processing Systems.

[50] Yi X., Walia E., Babyn P. (2018). Unsupervised and semi-supervised learning with Categorical Generative Adversarial Networks assisted by Wasserstein distance for dermoscopy image Classification. arXiv.

[51] Springenberg J.T. Unsupervised and semi-supervised learning with categorical generative adversarial networks. Proceedings of the International Conference on Learning Representations.

[52] Gulrajani I., Ahmed F., Arjovsky M., Dumoulin V., Courville A. (2017). Improved training of Wasserstein GANs. Proceedings of the 31st International Conference on Neural Information Processing Systems (NIPS’17).

[53] Gutman D., Codella N.C., Celebi E., Helba B., Marchetti M., Mishra N., Halpern A. Skin Lesion Analysis Toward Melanoma Detection: A Challenge at The International Symposium on Biomedical Imaging (ISBI) 2016, Hosted by The International Skin Imaging Collaboration (ISIC). Proceedings of the 2018 IEEE 15th International Symposium on Biomedical Imaging (ISBI 2018).

[54] Liu Y., Chen A., Shi H., Huang S., Zheng W., Liu Z., Zhang Q., Yang X. (2021). CT Synthesis from MRI Using Multi-Cycle GAN For Head-And-Neck Radiation Therapy. Comput. Med. Imaging Graph..

[55] Baur C., Albarqouni S., Navab N. MelanoGANs: High-resolution skin lesion synthesis with GANs. Proceedings of the 1st Conference on Medical Imaging with Deep Learning (MIDL2018).

[56] Yan S., Liu Y., Li J., Xiao H. DDGAN: Double Discriminators GAN for Accurate Image Colorization. Proceedings of the 2020 6th International Conference on Big Data and Information Analytics (BigDIA).

[57] Denton E.L., Chintala S., Szalm A., Fergus R. (2015). Deep generative image models using a Laplacian pyramid of adversarial networks. Adv. Neural Inf. Process. Syst..

[58] Fossen-Romsaas S., Storm-Johannessen A., Lundervold A.S. Synthesizing skin Lesion images using CycleGANs—A case Study. Proceedings of the NIK-2020 Conference.

[59] Karras T., Aila T., Laine S., Lehtinen J. Progressive Growing of GANs for Improved Quality, Stability, And Variation. Proceedings of the 6th International Conference on Learning Representations (ICLR 2018).

[60] Baur C., Albarqouni S., Navab N. (2018). Generating Highly Realistic Images of Skin Lesions with GANs. OR 2.0 Context-Aware Operating Theaters, Computer-Assisted Robotic Endoscopy, Clinical Image-Based Procedures, and Skin Image Analysis.

[61] Jiang M., Zhi M., Wei L., Yang X., Zhang J., Li Y., Wang P., Huang J., Yang G. (2021). FA-GAN: Fused Attentive Generative Adversarial Networks for MRI Image Super-Resolution. Comput. Med. Imaging Graph..

[62] Wang T., Liu M., Zhu J., Tao A., Kautz J., Catanzaro B. High-resolution image synthesis and semantic manipulation with conditional GANs. Proceedings of the IEEE Conference on Computer Vision and Pattern Recognition (CVPR).

[63] Beynek B., Bora Ş., Evren V., Ugur A. (2021). Synthetic Skin Cancer Image Data Generation Using Generative Adversarial Neural Network. Int. J. Multidiscip. Stud. Innov. Technol..

[64] Pang T., Wong J.H.D., Ng W.L., Chan C.S. (2021). Semi-supervised GAN-based Radiomics Model for Data Augmentation in Breast Ultrasound Mass Classification. Comput. Methods Programs Biomed..

[65] Shahsavari A., Ranjbari S., Khatibi T. (2021). Proposing a novel Cascade Ensemble Super-Resolution Generative Adversarial Network (CESR-GAN) method for the reconstruction of super-resolution skin lesion images. Inform. Med. Unlocked.

[66] Rashid H., Tanveer M.A., Khan H.A. Skin lesion classification using GAN-based data augmentation. Proceedings of the 41st Annual International Conference of the IEEE Engineering in Medicine & Biology Society (EMBC).

[67] Adhikari A. (2019). Skin Cancer Detection Using Generative Adversarial Network and an Ensemble of Deep Convolutional Neural Networks. Master’s Thesis.

[68] Tschandl P., Rosendahl C., Kittler H. (2018). The HAM10000 dataset: A large collection of multi-sources dermatoscopic images of common pigmented skin lesions. Sci. Data.

[69] Huang G., Liu Z., Weinberger K.Q. Densely connected convolutional networks. Proceedings of the 2017 IEEE Conference on Computer Vision and Pattern Recognition (CVPR).

[70] He K., Zhang X., Ren S., Sun J. Deep residual learning for image recognition. Proceedings of the 2016 IEEE Conference on Computer Vision and Pattern Recognition (CVPR).

[71] Mutepfe F., Kalejahi B.K., Meshgini (2021). S.; Danishvar, S. Generative Adversarial Network Image Synthesis Method for Skin Lesion Generation and Classification. J. Med. Signals Sens..

[72] Qin Z., Liu Z., Zhu P., Xue Y. (2020). A GAN-based image synthesis method for skin lesion classification. Comput. Methods Programs Biomed..

[73] Heenaye-Mamode Khan M., Gooda Sahib-Kaudeer N., Dayalen M., Mahomedaly F., Sinha G.R., Nagwanshi K.K., Taylor A. (2022). Multiclass Skin Problem Classification Using Deep Generative Adversarial Network (DGAN). Comput. Intell. Neurosci..

[74] Zhao C., Shuai R., Ma L., Liu W., Hu D., Wu M. (2021). Dermoscopy Image Classification Based on StyleGAN and DenseNet201. IEEE Access.

[75] Wei L.S., Gan Q., Ji T. (2018). Skin Disease Recognition Method Based on Image Color and Texture Features. Comput. Math Methods Med..

[76] Devaraj S.J. (2019). Emerging Paradigms in Transform-Based Medical Image Compression for Telemedicine Environment. Telemedicine Technologies.

[77] Zhu Y., Dai Y., Han K., Wang J., Hu J. (2022). An efficient bicubic interpolation implementation for real-time image processing using hybrid computing. J. Real-Time Image Proc..

[78] Rajarapollu P.R., Mankar V.R. (2017). Bicubic Interpolation Algorithm Implementation for Image Appearance Enhancement. Int. J. Comput. Sci. Technol..

[79] Nuno-Maganda M.A., Arias-Estrada M.O. Real-time FPGA-based architecture for bicubic interpolation: An application for digital image scaling. Proceedings of the 2005 International Conference on Reconfigurable Computing and FPGAs (ReConFig’05).

[80] Triwijoyo B., Adil A. (2021). Analysis of Medical Image Resizing Using Bicubic Interpolation Algorithm. J. Ilmu Komput..

[81] Yuan S., Abe M., Taguchi A., Kawamata M. (2007). High Accuracy Bicubic Interpolation Using Image Local Features. IEICE Trans. Fundam. Electron. Commun. Comput. Sci..

[82] Xie Y., Ning L., Wang M., Li C. (2019). Image Enhancement Based on Histogram Equalization. J. Phys. Conf. Ser..

[83] Gaddam P.C.S.K., Sunkara P. (2016). Advanced Image Processing Using Histogram Equalization and Android Application Implementation. Master’s Thesis.

[84] Atta M., Ahmed O., Rashed A., Ahmed M. (2021). Image Enhancement for Performance Improvement: Mathematics, Machine Learning and Deep Learning Solutions. Adv. Image Enhanc..

[85] Gonzalezand R.C., Woods R.E. (2008). Digital Image Processing.

[86] Wubuli A., Zhen-Hong J., Xi-Zhong Q., Jie Y., Kasabov N. (2014). Medical image enhancement based on shearlet transform and unsharp masking. J. Med. Imaging Health Inform..

[87] Reza A.M. (2004). Realization of the contrast limited adaptive histogram equalization (CLAHE) for real-time image enhancement. J. VLSI Signal Process. Syst. Signal Image Video Technol..

[88] Polesel A., Ramponi G., Mathews V.J. (2000). Image enhancement via adaptive unsharp masking. IEEE Trans. Image Process..

[89] Munadi K., Muchtar K., Maulina N., Pradhan B. (2020). Image Enhancement for Tuberculosis Detection Using Deep Learning. IEEE Access.

[90] Agaian S.S., Panetta K., Grigoryan A.M. (2001). Transform-based image enhancement algorithms with performance measure. IEEE Trans. Image Process..

[91] Nevils B., Mimbs T., Sailesh A., Naheed N. High Frequency Emphasis Filter Instead of Homomorphic Filter. Proceedings of the 2018 International Conference on Computational Science and Computational Intelligence (CSCI).

[92] Santhosh B., Rishikesan J., Sundar K., Kalaiyarasi M. (2021). Filters in Medical Image Processing. Suraj Punj. J. Multidiscip. Res..

[93] Rodríguez-Rodríguez J.A., Molina-Cabello M.A., Benítez-Rochel R., López-Rubio E. The effect of image enhancement algorithms on convolutional neural networks. Proceedings of the 2020 25th International Conference on Pattern Recognition (ICPR).

[94] Hasan M.K., Dahal L., Samarakoon P.N., Tushar F.I., Martí R. (2020). DSNet: Automatic dermoscopic skin lesion segmentation. Comput. Biol. Med..

[95] Hoshyar A.N., Al-Jumaily A., Hoshyar A.N. (2014). The Beneficial Techniques in Preprocessing Step of Skin Cancer Detection System Comparing. Procedia Comput. Sci..

[96] International Color Consortium Specification ICC.1: 2004-10 (Profile Version 4.2.0.0) Image Technology Colour Management—Architecture, Profile Format, and Data Structure, International Color Consortium, 2006, Revised 2019. https://www.color.org/icc_specs2.xalter.

[97] Al-saleem R.M., Al-Hilali B.M., Abboud I.K. (2020). Mathematical Representation of Color Spaces and Its Role in Communication Systems. J. Appl. Math..

[98] Ruslau M.F.V., Pratama R.A., Nurhayati, Asmal S. (2019). Edge detection in noisy images with different edge types. IOP Conf. Ser. Earth Environ. Sci..

[99] Church J.C., Chen Y., Stephen V., Rice A. (2009). Spatial Median Filter for Noise Removal in Digital Images.

[100] Rajlaxmi Chouhan C., Pradeep K.C., Kumar R. (2012). Contrast enhancement of dark images using stochastic resonance in the wavelet domain. Int. J. Mach. Learn. Comput..

[101] Janani P., Premaladha J., Ravichandran K.S. (2015). Image Enhancement Techniques: A Study. Indian J. Sci. Technol..

[102] Yadav S.S., Jadhav S.M. (2019). Deep convolutional neural network based medical image classification for disease diagnosis. J. Big Data.

[103] Wang J., Yang Y., Wang T., Sherratt R.S., Zhang J. (2020). Big data service architecture: A survey. J. Internet Technol..

[104] Liu B., Lv J., Fan X., Luo J., Zou T. (2022). Application of an Improved DCGAN for Image Generation. Mob. Inf. Syst..

[105] Zhong G., Gao W., Liu Y., Yang Y. (2020). Generative Adversarial Networks with Decoder-Encoder Output Noise. Neural Netw..

[106] Nilsson J. (2020). Understanding SSIM. arXiv.

[107] Behara K., Bhero E., Agee J.T., Gonela V. (2022). Artificial Intelligence in Medical Diagnostics: A Review from a South African Context. Sci. Afr..

[108] Ghassemi N., Shoeibi A., Rouhani M. (2020). Deep neural network with generative adversarial networks training for brain tumour classification based on MR images. Biomed. Signal Process. Control.

